# Cyanobacterial membrane dynamics in the light of eukaryotic principles

**DOI:** 10.1042/BSR20221269

**Published:** 2023-02-23

**Authors:** Carmen Siebenaller, Dirk Schneider

**Affiliations:** 1 Department of Chemistry, Biochemistry, Johannes Gutenberg University Mainz, 55128 Mainz, Germany; 2 Institute of Molecular Physiology, Johannes Gutenberg University Mainz, 55128 Mainz, Germany

**Keywords:** cyanobacteria, dynamin-like protein, membrane biogenesis, membrane dynamics, thylakoid membrane, Vipp1

## Abstract

Intracellular compartmentalization is a hallmark of eukaryotic cells. Dynamic membrane remodeling, involving membrane fission/fusion events, clearly is crucial for cell viability and function, as well as membrane stabilization and/or repair, e.g., during or after injury. In recent decades, several proteins involved in membrane stabilization and/or dynamic membrane remodeling have been identified and described in eukaryotes. Yet, while typically not having a cellular organization as complex as eukaryotes, also bacteria can contain extra internal membrane systems besides the cytoplasmic membranes (CMs). Thus, also in bacteria mechanisms must have evolved to stabilize membranes and/or trigger dynamic membrane remodeling processes. In fact, in recent years proteins, which were initially defined being eukaryotic inventions, have been recognized also in bacteria, and likely these proteins shape membranes also in these organisms. One example of a complex prokaryotic inner membrane system is the thylakoid membrane (TM) of cyanobacteria, which contains the complexes of the photosynthesis light reaction. Cyanobacteria are evolutionary closely related to chloroplasts, and extensive remodeling of the internal membrane systems has been observed in chloroplasts and cyanobacteria during membrane biogenesis and/or at changing light conditions. We here discuss common principles guiding eukaryotic and prokaryotic membrane dynamics and the proteins involved, with a special focus on the dynamics of the cyanobacterial TMs and CMs.

## Introduction

Often, a simple definition is used to separate pro- from eukaryotes: as the greek meaning indicates (*eu:* ‘good’ and *karyon:* ‘core’ or ‘nucleus’), the DNA of eukaryotes is encapsulated in a membrane-surrounded cellular compartment, the nucleus, whereas the DNA of prokaryotes (*pro:* ‘before’) is located freely within the cytoplasm. Furthermore, a complex cell structure and intracellular organization are also believed to be features that specifically characterize eukaryotes. Examples of this cellular complexity are compartmentalization by the formation of membrane-enclosed organelles, an intracellular-trafficking system, or the uptake of external substances by endocytosis [1–3]. While eukaryotes are undeniably more complex in many aspects, a certain level of complexity is present in prokaryotes as well [4], and the recent identification of compartmentalized bacteria that might even contain a primitive nucleus clearly challenges our traditional view on the clear-cut separation of pro- from eukaryotes [5]. This is also true on the level of cellular functions, and some proteins originally identified and termed as *eukaryotic signature proteins* [6] actually have archaeal or bacterial homologs, demonstrating that the eukaryotic machineries have already developed in an earlier stage of evolution in a prokaryotic ancestor and are no real eukaryotic inventions [7–9]. Several of these proteins are involved in mediating and/or regulating the dynamics of intracellular membranes, in pro- as well as eukaryotes.

### Internal membranes are present in eu- as well as prokaryotic cells

The cytoplasmic membranes (CMs) of prokaryotic and eukaryotic cells are selective barriers that separate the inside from the outside world of a cell. The chemical composition, structure, and biophysical properties of eukaryotic and bacterial CMs are in general similar, while they can differ substantially from archaeal membranes [10]. Typical membranes are based on lipid bilayers, contain transmembrane proteins as well as proteins bound at their surfaces and are thin, fluid, and flexible. The biophysical properties of membranes allow them to fuse and/or to pinch off vesicles. All membranes consist of diverse lipid species, which are partially conserved in eukaryotes and bacteria, but also in some cases highly specific for defined domains of life, organisms, and/or organelles [11,12]. The main membrane lipid species of eukaryotes are sphingolipids, sterols, and phosphoglycerolipids, while most bacterial membranes in general mainly consist of phosphoglycerolipids. These lipids typically serve as a paradigm for the membrane lipid structure and biophysical properties: two fatty acids, saturated and/or nonsaturated, are bound via an ester linkage to a glycerol backbone. At the C3 position, a phosphate is bound, to which diverse polar head groups are attached that contain a hydroxyl group, resulting in an amphiphilic molecule [13]. Yet, the lipid composition of chloroplast and cyanobacterial membranes differs from other eu- and prokaryotic membranes, as usually less than 15% of the membrane lipids belong to the class of phosphoglycerolipids. The chloroplast and cyanobacterial membranes predominantly consist of the phosphorous-free galactolipids monogalactosyldiacylglycerol (MGDG), digalactosyldiacylglycerol (DGDG) and sulfoquinovosyldiacylglycerol (SQDG), where the sugar moieties are directly linked to the glycerol backbone [14–16].

Besides the cell-surrounding CM, further additional internal membrane systems are present in eukaryotes and some prokaryotes. In eukaryotes, internal organelles are surrounded by membranes that separate defined reaction compartments from the cytoplasm. Examples of prokaryotic internal membrane systems are the thylakoid membranes (TMs) in cyanobacteria [17], the chromatophore membrane in purple phototrophic bacteria [18], magnetosoms of magnetotactic bacteria [19], or the internal membrane systems observed in methane-oxidizing bacteria [20,21]. Recently, also a clearly defined, membrane-enclosed vacuole and (likely) membrane-enclosed DNA, a primitive nucleus, have been observed in a bacterium [5]. Actually, just a few proteins within a prokaryote can already induce defined internal membrane structures. The heterologous expression of the two *Vibrio cholerae* proteins CrvA and CrvB already generated membrane asymmetry in *Agrobacterium tumefaciens* [22], and heterologous expression of the *Shewanella oneidensis* MR-1 BAR domain protein in *Escherichia coli* or *Marinobacter atlanticus* resulted in the formation of outer membrane extensions [23].

### Dynamic membrane organization is vital in eu- as well as prokaryotes

Membranes do not only define cell boundaries and compartments but also separate regions of differing composition and enable cells to maintain electric and/or chemical gradients. Nevertheless, membranes are selectively permeable and (actively) transport molecules and/or ions. Clearly, preserving the membrane structure and repair of damaged membrane regions is crucial in pro- and eukaryotes to maintain the membrane’s barrier function as well as cellular compartmentalization. Yet, under physiological conditions membranes are not in equilibrium and are constantly remodeled with respect to lipid and transmembrane protein distribution as well as the membrane shape. The term ‘membrane dynamics’ is here used to summarize all these processes. Examples of such processes are cell division or vesicular transfer processes in eukaryotes.

The vesicular transport in the secretory and/or endocytic pathways involves the formation of vesicular structures and their subsequent fusion with target membranes. While vesicle formation requires the induction of highly curved membrane regions at the donor membrane, which finally results in vesicle budding, these curved vesicles then need to fuse with the usually flat surface of a target membrane [24]. Both processes involve extensive remodeling of the lipid bilayer structure. A different example of dynamic membrane remodeling observed in eukaryotes is the complete disassembly and reassembly of the nuclear envelope during cell division [25]. Reassembly of the nuclear envelope membranes requires the coordinated formation of the membrane system by fusion of vesicular/tubular structures as well as membrane integration of transmembrane proteins [25,26]. Yet, although bacteria typically have fewer internal membrane structures, membrane remodeling of the CM is also vital in several cases, including cell division, cell motility, or sporulation [4]. Furthermore, membrane remodeling is relevant in more specialized bacterial internal membrane systems, such as the TMs of cyanobacteria, which will be discussed in detail below. In general, the membrane structure of any organism eventually adapts to certain physiological conditions, and thus, the membrane structure and organization need to be flexible and dynamic.

The dynamics of membrane systems are determined by several factors: First, the lipid composition and distribution have decisive effects on the biophysical properties of the membrane. While bilayer-forming lipids typically have a cylindrical shape, which allows the formation of a stable, lamellar lipid bilayer structure, so-called non-bilayer-forming lipids have a conical shape that destabilizes the traditional membrane organization [27]. Notably, membranes of prokaryotic origin are typically rich in non-bilayer-forming lipids, whereas eukaryotic membranes mainly contain membrane-stabilizing lipid species [11,13]. Yet, non-bilayer-forming lipids, such as phosphatidylethanolamine (PE) or cardiolipin, are often involved in dynamic membrane-remodeling processes, such as membrane fusion at the Golgi membrane (PE) or the fusion of mitochondria (cardiolipin), but also in the formation of intracellular membranes in bacteria [28,29]. Furthermore, the length and saturation of the lipid acyl chains influence the membrane lipid order, and the lipid head group chemistry potentially affects interactions with proteins or different membranes. Membrane dynamics can thus be regulated by controlled biosynthesis or the spatiotemporal accumulation of defined lipids within distinct membranes or membrane regions.

Besides the lipids, also soluble, peripherally attached or transmembrane proteins might influence membrane dynamics. For instance, cytoskeletal proteins, such as actin or tubulin, determine the general architecture of a eukaryotic CM, and thus the shape of the entire cell. In fact, the actin cytoskeleton undergoes drastic rearrangement during cell division [30]. Membrane-interacting proteins can either passively organize a membrane (e.g., via crowding) or actively remodel membranes via triggering membrane bending, fusion or fission by energy-dependent structural rearrangements [31].

In the previous decades, membrane dynamics has been considered to be of greater importance in eukaryotes but less important (or absent) in prokaryotes, and thus, many underlaying biophysical principles and the proteins involved have initially been identified, described and studied in eukaryotes. Only in the recent decade, it became obvious that proteins triggering membrane dynamics are also present in bacteria, where, however, their exact physiological function often is not finally resolved yet. In the following, we present examples of protein families involved in membrane dynamics with common features in eu- and prokaryotes, and especially focus on cyanobacteria, prokaryotes with an extended internal membrane system.

### The complex internal membrane systems of cyanobacteria

Cyanobacteria are oxygenic photoautotrophic bacteria, and the chloroplasts of algae and plants presumably evolved in an endocytic event from an ancient cyanobacterium [32]. Consequently, as chloroplasts, also cyanobacteria contain two internal membrane systems: the CM, which corresponds to the inner envelope of chloroplasts, and the TM, a completely separated internal membrane system that harbors the complexes of the photosynthetic electron transfer chain [33]. The TM is protein-rich, and the protein complexes of the photosynthetic light reaction are present in plant as well as cyanobacterial TMs. Furthermore, in cyanobacteria, the components of the respiratory electron-transfer chain are also localized within the TMs [34]. During the photosynthetic light reaction, light energy is first collected by light-harvesting protein-pigment complexes at the TM and subsequentely converted into chemical energy (NADPH and ATP). In the electron-transfer chain, protons are pumped into the thylakoid lumen, resulting in a lowered pH compared to the cytoplasm, and this ΔpH is used for ATP production [35].

Besides a conserved function, the exact architecture of the TM network differs quite remarkably between chloroplasts and cyanobacteria, but also within different cyanobacterial species. The TMs of chloroplasts are typically separated into thylakoid stacks (grana thylakoids) and connecting, unstacked stroma thylakoids [36,37]. In contrast, most cyanobacteria form less complex TM systems with long, flat membranes, but also partially highly curved TM margins. In the to date best-studied cyanobacterium *Synechocystis* sp. PCC 6803 (hereafter: *Synechocystis*), the TMs form parallel uncurved membrane structures close to the CM, where they finally converge and likely form so-called thylapse structures [38], whereas cyanobacteria of the genus *Synechococcus* appear not to form such highly curved membrane regions [39]. In contrast with most other cyanobacteria, cyanobacteria of the genus *Gloeobacter*, such as *Gloeobacter violaceus PCC 7421* (hereafter: *Gloeobacter*), do not contain an extra internal TM system [40]. Within the *Gloeobacter* CM, the complexes of the photosynthetic electron-transfer chain appear to exist within defined lipid microdomains, and thus the *Gloebacter* CM appears to be laterally organized [40,41]. A laterally heterogenous organization of the TM has been observed also in other cyanobacteria, and distinct regions appear to have special functions: while some regions predominantly contain either photosystem I (PSI) or PSII [42,43], others appear to contain especially high amounts of ribosomes [38,44]. These later regions likely correspond to defined protein biosynthesis and/or repair zones [45–47], reminiscent of specialized TM *translation zones* observed in algal chloroplasts [48]. A lateral (re)organization of TM protein complexes is necessary to regulate efficient light harvesting and electron transport by adjusting the relative activities of PSI and PSII, which likely includes local separation of PSI and PSII in the membrane and the movement of soluble light-harvesting complexes [49,50].

The structure of TMs is highly dynamic in chloroplasts and cyanobacteria and develops, rearranges, and adapts to environmental changes, such as changing light conditions [51,52]. While the TM system of chloroplasts can develop in undifferentiated proplastids via vesicle release from the plastid inner envelope membrane and subsequent vesicle fusion [37,53], cyanobacterial TMs appear to develop as parallel stacks close to the CM most likely not completely *de novo* but from preexisting TM remnants [54]. This becomes, e.g., evident when cyanobacterial cells are grown at conditions where TMs are largely degraded, such as in the dark [52] or under conditions causing chlorosis [55,56]. Under changing light conditions, the TM network in plant chloroplast substantially rearranges, involving membrane fusion and fission events [57]. Furthermore, in the light, the TM is highly vulnerable to damage, since reactive oxygen species are generated in the photosynthetic light reaction, which eventually damage lipids and/or proteins [58]. Hence, besides membrane remodeling, membrane protection and repair mechanisms are urgently needed in TM-containing cells and organelles.

Clearly, TM biosynthesis and development require the coordinated synthesis and assembly of the individual membrane components, involving lipids, pigments, and proteins. The assembly might include transport of these components from the cyanobacterial CM or the chloroplast IE, respectively, to a preexisting TM structure or between individual TM stacks, either via a vesicular transport, as observed during chloroplast development [59–61], or via direct TM-CM/TM connections [62,63]. While an involvement of some proteins, such as the cyanobacterial PSII assembly factors PratA [64,65], the major lipid biosynthesis proteins MGD1/DGD1/MGS [66,67], the chloroplast THF1 [68], or Hsp70 proteins [69,70] has been described, the mechanisms guiding TM biogenesis in chloroplasts and cyanobacteria are still not understood in detail. Yet, one key player appears to be the i*nner membrane-associated protein of 30 kDa* (IM30)/*vesicle-inducing protein in plastids 1* (Vipp1), which is discussed in detail below as a protein involved in membrane remodeling.

**Figure 1:**
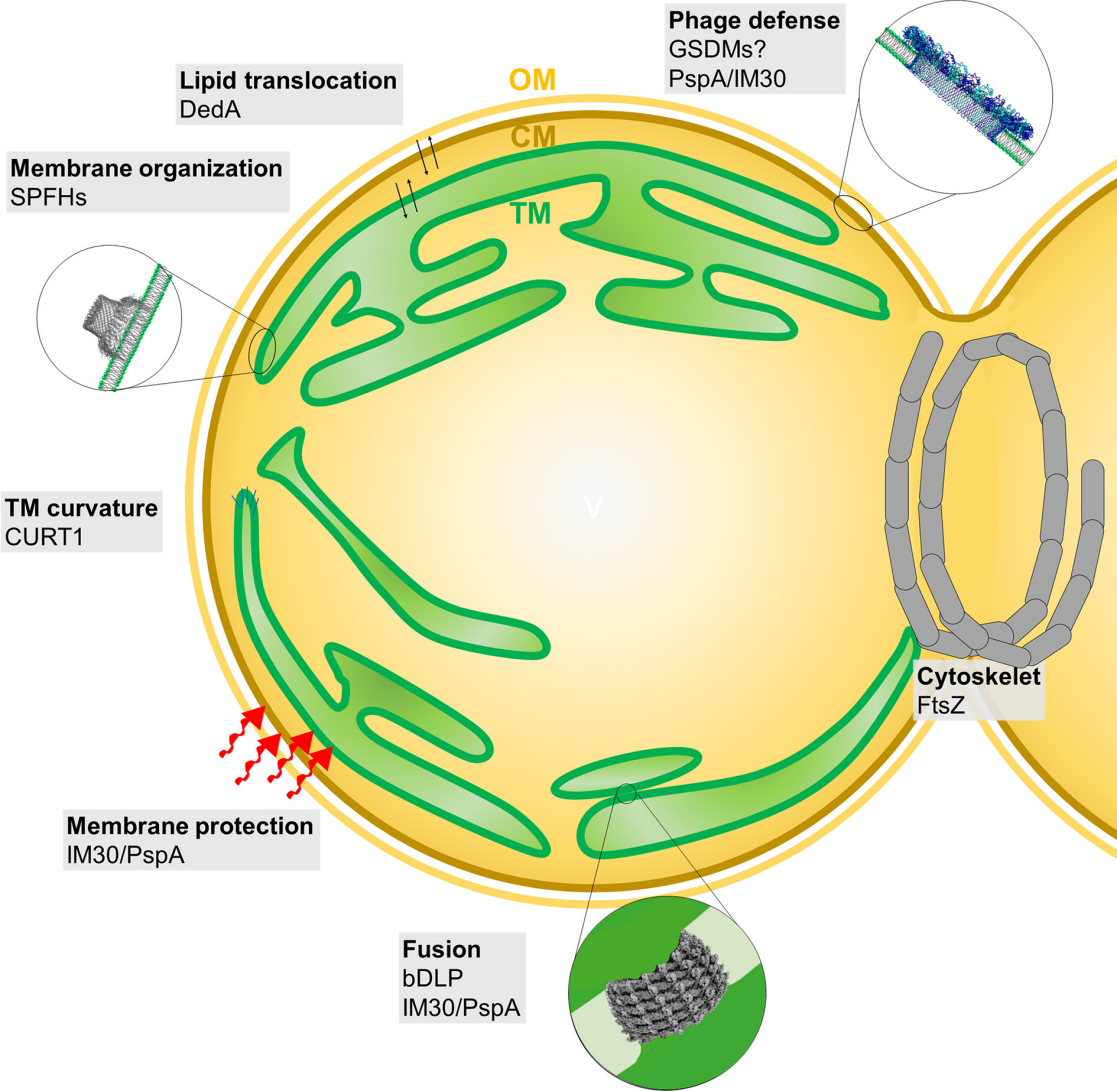
Proteins involved in cyanobacterial membrane dynamics Cyanobacterial TMs develop from TM remnants and dynamically remodel, e.g., during cell division, during acclimation to changing physiological conditions as well as under stress conditions. Membrane stabilization, destabilization, and/or dynamic membrane remodeling involves proteins that either stabilize membrane structures (Curvature thylakoid 1 [CURT1], IM30/phage shock protein A [PspA]), organize a membrane via lateral microdomain formation (SPFHs) or transversal lipid rearrangement (DedA), destabilize membrane via pore formation (GSDMs) or mediate membrane fusion (bDLPs, IM30). The structure and function of these proteins, as well as their putative involvement in cyanobacterial membrane dynamics, are discussed in the text.

In summary, for organizing, structuring, remodeling, stabilizing, and repairing cyanobacterial membranes, clearly diverse proteins are required, such as membrane fusion/fission proteins, lipid translocases, as well as membrane stabilizers and organizers. Unfortunately, the knowledge about such proteins in cyanobacteria is still limited. Yet, in recent years, several proteins involved in cyanobacterial membrane dynamics have been identified and characterized, many of which are homologs of eukaryotic proteins (Figure 1). Thus, their modes of action are likely based on similar principles. Interestingly, while in cell biology simpler prokaryotic homologs are frequently used to study and describe the structure and activity of eukaryotic proteins and protein complexes, here the eukaryotic proteins have often been studied more intensively yet. Consequently, potential functions of the (cyano)bacterial homologs are derived from and discussed within the context of the eukaryotic homologs.

## Structuring internal membranes via lipid asymmetry

Many membrane properties, such as fluidity, thickness, phase behavior, permeability, lipid–protein interactions, and stability, are largely defined by the specific lipid composition of the membrane. Therefore, any protein that is involved in lipid metabolism, such as acyltransferases, desaturases, or lipases, can affect the general membrane properties, which has been discussed for plant membranes in detail recently [71]. Since membrane lipids are not necessarily synthesized within the final target membrane or in the correct bilayer leaflet, systems to transport lipids across a bilayer are required to prevent lipid asymmetry. On the other hand, certain cellular functions may require lipid asymmetry, which can affect bilayer properties, such as the membrane surface charge, the membrane potential, as well as stability, permeability, and the membrane shape [72,73]. While much better studied and understood in eukaryotes, this ‘transversal’ lipid asymmetry has also been observed in bacteria [74].

Spatial accumulation of lipids in defined membrane areas (= lateral lipid asymmetry), i.e., the formation of lipid domains by which special reaction compartments are generated within membranes, has been observed in eukaryotes as well as in some bacteria [75,76]. Recently, the reversible formation of fluid *vs*. gel phases in living bacteria has been described, a putative mechanism enabling bacterial cells to spatiotemporarily assemble defined platforms, e.g., used for signaling [77]. The lateral asymmetry of lipids and proteins has been suggested to organize the CM of the cyanobacterium *Gloeobacter* [40] and potentially also triggers segregation of PSs into defined membrane regions, as has been observed in the TM-containing cyanobacteria *Synechocystis* and *Synechococcus elongatus* sp. PCC 7942 (hereafter: *Synechococcus elongatus*) [43,78,79].

### Transversal lipid asymmetry

While lateral lipid diffusion within one bilayer is fast, transversal lipid exchange between the leaflets is slow, as it is energetically disfavored to move the hydrophilic lipid headgroup across the hydrophobic membrane core region. Proteins, such as flippases, floppases, or scramblases, transport lipids across the membrane, even against a gradient. While flippases and floppases are ATP-dependent proteins, scramblases are energy-independent and only equilibrate lipids between the outer and inner leaflets of a membrane along a gradient [80].

Examples of flippases are the eukaryotic P_4_-type ATPases, which translocate lipids inwardly directed, while outwardly directed floppases typically belong to the evolutionary conserved ABC superfamily [81]. Flippases have been shown to be involved in various membrane-remodeling processes in eukaryotes, e.g., by inducing membrane curvature via local accumulation of nonbilayer-forming lipids in one membrane leaflet [82,83]. Pro- and eukaryotic flippases are homologous and have similar substrates, yet sometimes show a specificity for defined lipids. The bacterial flippase MprF mediates translocation of amino acid-modified lipids into the outer leaflet of the CM in some bacteria [84], and the flippase MurJ translocates a precursor lipid synthesized at the cytoplasmic leaflet of the CM across the membrane for cell wall synthesis [85]. While the outer membrane of Gram-negative bacteria is highly asymmetric, observations indicating a transversal asymmetry in the CM of Gram-negative bacteria are rare, and thus this issue still is controversially discussed [86]. Yet, recent analyses suggest that not only the CM lipids of Gram-positive bacteria but also of Gram-negative bacteria, such as *E. coli*, are distributed asymmetrically [87]. Nevertheless, specific flippases and/or floppases crucial for generation and maintenance of a transversal lipid asymmetry in bacterial CMs are not described yet, to the best of our knowledge.

Besides flippases and floppases, also scramblases can be involved in remodeling of curved membranes. When, e.g., the curvature of a membrane results from asymmetrically distributed lipids, a scramblase activity can lead to flattening of curved membranes by equilibration of the two bilayer leaflets [88]. The scramblase activity might also inhibit extensive bending of flat membranes, when nonbilayer forming lipids, such as PE or MGDG, are synthesized at one side of the membrane. This appears to be especially important in cyanobacterial membranes, where large amounts of the non-bilayer-forming lipid MGDG are present and need to be balanced with bilayer-forming lipids, such as DGDG [89]. Interestingly, the MGDG/DGDG ratio correlates with the curvature of the TMs, at least in chloroplasts [90]. While a transversal lipid asymmetry has not yet been described specifically for the cyanobacterial CM or TM, the recent observation of CM asymmetry in some bacteria might indicate that also bacterial inner membrane systems more generally show transversal lipid asymmetry.

**Figure 2:**
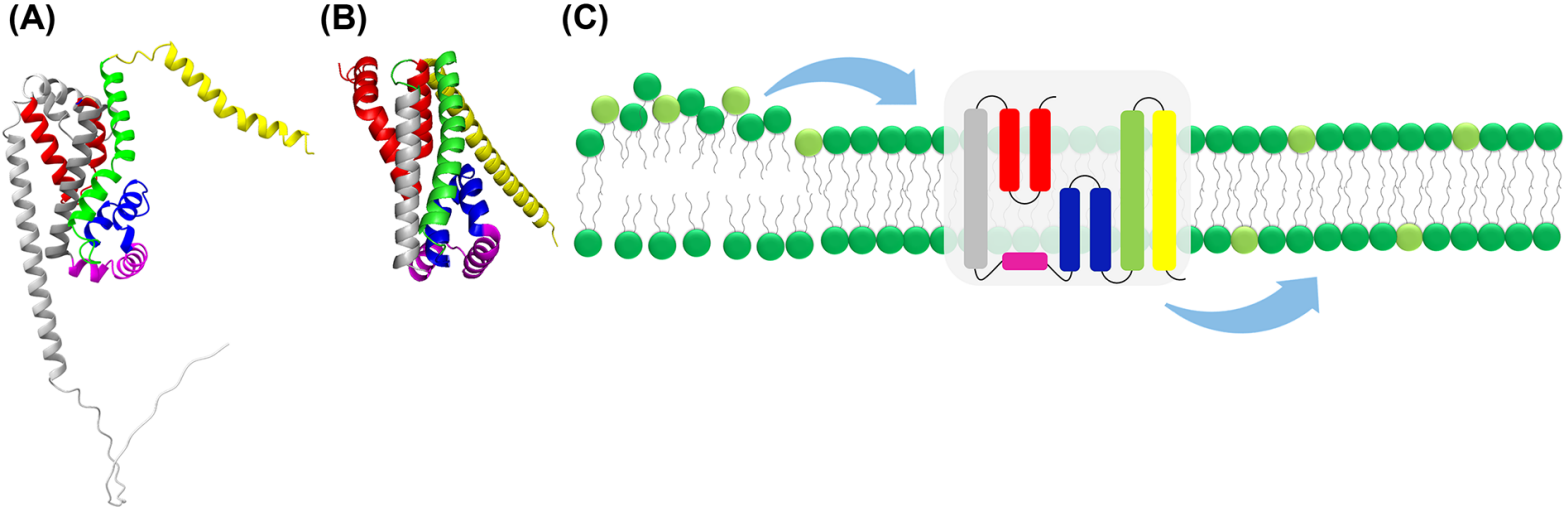
Putative structure and function of cyanobacterial DedA proteins AlphaFold (106,107) models of the DedA proteins (**A**) TMEM41B (human) and (**B**) Slr0232 (*Synechocystis*) suggesting a conserved structure. The proteins are predicted to be mainly α-helical with two reentrant loops facing each other in the membrane core. (**C**) We suggest that also the cyanobacterial DedA proteins have scramblase activity to reduce lipid asymmetry in the CM and/or TM, e.g., resulting from localized lipid biosynthesis.

An example of scramblases conserved in eu- and prokaryotes are proteins of the DedA (*downstream of hisT* E. coli *DNA geneA*) family. The DedA protein superfamily is conserved in all kingdoms of life, and consists of the VMP1, TMEM41, DedA, and the PF066095 family [91]. While eukaryotes typically contain only proteins of the VMP1 and TMEM41 family, representatives of all four families are present in prokaryotes [91]. Unfortunately, the physiological function of these proteins is only poorly understood thus far. Yet, the family members share a common structure, which typically consists of 4–6 transmembrane helices [91] (Figure 2). Thus far, no experimentally derived structure of any DedA protein is available. The DedA domain of human TMEM41b is predicted to form two membrane-spanning helices, one extramembrane helix and two reentrant loops, which face each other in the membrane core [92,93] (Figure 2).

The eukaryotic DedA proteins were initially described being involved in vesicle formation and/or transport in the secretory pathway. The DedA protein Tvp38 of *Saccharomyces cerevisiae* has been reported to interact with the T-SNARE complex [94], but relevance for the SNARE function has not been demonstrated *in vivo* thus far [95]. Recently, the eukaryotic DedA proteins TMEM41B and VMP1 have been shown to have scramblase activity [96–98]. These proteins are located within the ER membrane and are suggested to regulate various dynamic membrane processes, such as the formation of autophagosomes or lipoprotein particles [92,96].

In the bacterium *E. coli*, eight different DedA proteins are encoded, which are individually not essential, albeit deletion of all protein-coding genes resulted in a lethal phenotype [99]. In *Borrelia burgdorferi*, only one DedA protein is expressed, which is essential [100]. Deletion of bacterial DedA resulted in cell division defects, altered lipid composition and a loss of the proton motive force [101–103]. As deletion of the *E. coli*, DedA subfamily members YqjA and YghB resulted in altered phospholipid levels [99], the scramblase activity might be conserved within the whole DedA superfamily [92], and may underlie the various membrane-associated functions attributed to DedA superfamily proteins.

Tvp38 homologs have also been identified in chloroplasts and in cyanobacteria [104]. While chloroplasts typically only encode one Tvp38/DedA protein [105], multiple proteins of this family appear to be conserved in cyanobacteria. Three DedA proteins are encoded in *Synechocystis* (Table 1). Thus far, the exact structure and function of these proteins are unknown, yet, based on the assumed functions described in other bacteria, involvement of these proteins in TM and/or CM homeostasis and/or dynamics appears to be likely. While a transversal lipid asymmetry has not been described in cyanobacterial membranes, *de novo* synthesis of cyanobacterial lipids will result in an asymmetric lipid distribution, i.e., accumulation of defined lipid species within only one leaflet of the lipid bilayer. As this clearly bears the risk of a lipid-mediated membrane remodeling (as discussed above), scramblases of the DedA protein family are potentially involved in reducing lipid asymmetry in cyanobacterial membranes (Figure 2).

**Table 1:** *Synechocystis* proteins with homology to eukaryotic proteins (putatively) involved in membrane dynamics

*Synechocystis* protein^1^	Eukaryotic homolog	(Putative) functions in eukaryotic cells
**DedA proteins**		
Slr0232	Tvp38	Vesicular trafficking, putative lipid scramblase
Slr0305		
Sll0509		
**SPFH proteins**		
Slr1106	Prohibitin	Stabilization and dynamics of mitochondrial membrane, associated with lipid rafts
Slr1768		
Sll1021	Flotillin	Associated with lipid rafts
Slr1128	Stomatin	Ion channel regulation, associated with lipid rafts
Sll0815		
**Cytoskeletal proteins**		
Sll1633 (FtsZ)	Tubulin	Microtubule formation
**Membrane-bending proteins**		
Slr0483	*Arabidopsis* CURT1	Induction of curvature in TM
**Dynamin-like proteins**		
Slr0869	Dynamin	Membrane fission/fusion
**ESCRT-III proteins**		
Sll0617 (IM30)	ESCRT-III	Membrane fission
Slr1188 (PspA)		

1 The protein nomenclature follows the CyanoBase database [256].

### Lateral lipid asymmetry

TMs appear to be a largely homogeneous lipid mixture, and formation of defined lipid domains is not observed in simulations in absence of proteins. Yet, even in the simulations, nonideal lipid mixing has been observed, most notably the clustering of PG lipids and of lipids with either fully saturated or fully polyunsaturated acyl tails [108]. Thus, the basic membrane constituents, the membrane lipids, may already form segregated lipid domains with defined physicochemical properties, albeit such domains are likely stabilized by protein components in cyanobacteria. With respect to the protein distribution, the cyanobacterial TM appears to be heterogeneous, and membrane microdomains appear to exist in TMs where defined proteins and protein complexes locally accumulate [35,43,79].

While it currently still is largely enigmatic how such defined lipid micro- or nanodomains form in cyanobacterial TMs, proteins of the SPFH superfamily were found to be enriched in lipid domains observed in pro- and eukaryotes under defined conditions. These domains typically contain increasing amounts of sphingolipids or cholesterol in eukaryotes and increasing amounts of polyisoprenoid lipids in some prokaryotes [109–111]. The SPFH protein superfamily consists of the protein families stomatin, prohibitin, flotillin and the bacterial HflK/C. Members of this superfamily are widespread among all domains of life. Most likely, the eukaryotic subfamilies evolved from the respective prokaryotic subfamilies, which lastly all appear to have a common prokaryotic origin [112]. A feature common to all superfamily members is the conserved SPFH domain, which consists of four α-helices and a twisted β-sheet structure [113]. All members of this superfamily appear to form large, membrane scaffolding, partially membrane-anchored oligomeric structures [109,110].

SPFH proteins have multiple physiological functions, which are highly specific in some cases. Most likely, the cellular function is determined by the specific interaction of the SPFH proteins with other proteins present within SPFH-defined lipid domains. In eukaryotic membranes, these functions include CM organization, cytoskeletal rearrangement, signal transduction, endocytosis, or chromosome segregation during cytosis [114,115]. Prohibitines are mainly located in the inner membrane of mitochondria [116], where they appear to be involved in mitochondrial membrane stabilization and dynamics, albeit they appear also to be crucial for cell proliferation [117]. Stomatines are discussed to regulate the activity of ion channels, such as acid-sensing ion channels in vertebrate neurons, or transporters, such as GLUT-1 [118,119]. More precisely, the function of stomatines in eukaryotes might be the recruitment of microdomain-specific lipids, such as cholesterol [120,121]. Eukaryotic flotillin is associated with vesicular trafficking and signal transduction, yet its exact physiological function remains largely unclear.

**Figure 3:**
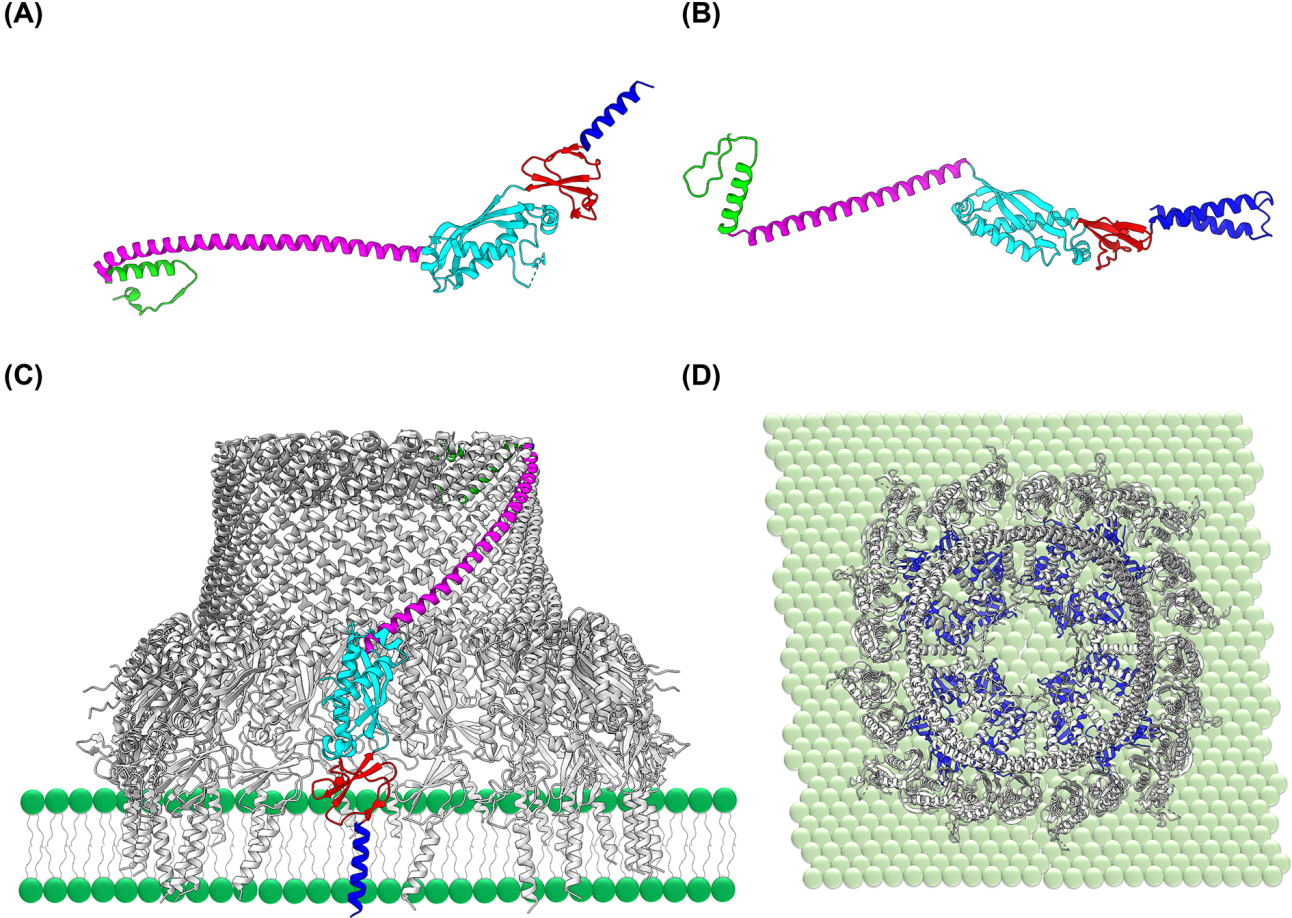
Structure and membrane interaction of the *E. coli* HflK/C (complex) and the *Synechocystis* SPFH protein Slr1768 (**A**) The structure of the monomeric *E. coli* SPFH protein HflK (pdb 7WI3) as well as (**B**) the AlphaFold (106,107) predicted structure of the *Synechocystis* Slr1768 protein. In (**C**), the structure of the *E. coli* HflK/C complex is shown (pdb 7WI3). (**B**) The protein Slr1768 of the cyanobacterium *Synechocystis* is predicted to have a typical HflK/C protein structure (106,107). (**C,D**) The *E. coli* HflK/C complex is partially integrated into the membrane and encloses a distinct membrane area. HflK and HflC with their transmembrane helices act like a fence with fence posts. (**D**) Within this *E. coli* complex, the FtsH protease (blue) is located. In cyanobacteria, the encoded SPFH proteins likely also form laterally enclosed, clearly defined membrane regions within the TM and/or the CM.

HflK/C, the bacterial homologs of the human prohibitines, are located within the bacterial CM. Recently, the structure of a bacterial lipid domain formed by *E. coli* HflK and HflC proteins in complex with the membrane-anchored AAA+ protease FtsH has been determined via high-resolution cryo-EM [122]. Here, the transmembrane domains of HflK and HflC form an oligomeric, circular assembly, and completely seal a lipid domain, in which four FtsH hexamers were located (Figure 3). Likely, HflK/C regulate the FtsH activity via the formation of such defined reaction compartment. This membrane segregation and lipid domain formation by bacterial prohibitins is probably not only relevant for HflK/C proteins and FtsH but rather serves as a general mechanism of microdomain formation and activity regulation by SPFH proteins.

Besides prohibitines, also flotillines are present in bacteria. In *Bacillus subtilis*, the flotillins FloA and FloT are discussed to functionally organize the bacterial membrane via the formation of microdomains [123], which appear to be important for transmembrane signal transduction and transport processes. The *B. subtilis* flotillin YdjH has been shown to be involved in the recruitment of the bacterial *endosomal sorting complexes required for transport*-III (ESCRT-III) protein PspA (discussed later) to membranes, which is part of a membrane stress response system involved in bacterial membrane protection [124]. Noteworthy, expression of the genes coding for *B. subtilis* flotillines is genetically regulated by the stress-responsive ECF sigma factor σ^w^, indicating that flotillines also have stress-responsive, membrane-remodeling, and/or -stabilizing functions rather than just being involved in the formation of defined membrane domains [125]. While also stomatines are clearly present in bacteria, their function remains unclear, similar to the situation in eukaryotics.

SPFH proteins are also encoded in cyanobacteria. Five SPFH proteins have been identified in *Synechocystis* (Table 1), with two members belonging to the prohibitin/HflC/K family (Slr1106, Slr1768), one flotillin homolog (Sll1021), and two stomatin homologs (Slr1128, Sll0815) [126,127]. In contrast, only one prohibitin and one stomatin homolog are encoded in the genome of the cyanobacterium *Thermosynechococcus vestitus* BP-1 (formerly *Thermosynechococcus elongatus* BP-1) [126]. To date, 3121 proteins of 664 cyanobacterial species are listed in the InterPro database being members of the SPFH superfamily (IPR 036013; accessed November 2022)). Approximately 350 cyanobacterial species contain flotillins (IPR 027705), ∼600 species contain prohibitines (IPR 000163), and ∼550 species stomatines/HflKs (IPR 001972).

Some *Synechocystis* SPFH proteins were found to be associated with the CM (Slr1106, Slr1768, and Slr1128), whereas the *Synechocystis* prohibitin/HflK/C Slr1106 binds to TMs [126]. While for most superfamily members, no decisive involvement of the proteins in TM biogenesis and/or maintenance has been identified [126], the prohibitin Slr1768 appears to regulate the maintenance of TMs, especially upon light-induced damage [127]. Yet, Slr1768 (Figure 3) has initially been identified to be associated with the *Synechocystis* CM [126], and thus, the protein might have a dual membrane localization and/or is located in between the two internal membrane systems in cyanobacteria. Also for the stomatin homolog Slr1128, which has initially been identified to be associated with the CM, it has been suggested that the protein is located at the CM/TM interface in specialized regions crucial for PS biogenesis [45,128]. In fact, the stomatin homolog Slr1128 as well as the prohibitin homolog Slr1106 appear to associate with PSII subunits together with the FtsH2/3 protease [128]. Thus, at least some cyanobacterial SPFH superfamily members appear to be involved in the formation of defined lipid domains crucial for the biogenesis and/or repair of PSII. In such domains, the cyanobacterial SPFH proteins might be responsible for the regulation of the FtsH protease activity [128], consistent with the FtsH and HflC/K interaction identified in *E. coli*, as discussed above. In line with this assumption, at least the *Synechocystis* stomatin homolog Slr1128 forms large prototypical ring structures, the basis for membrane organization by SPFH superfamily members [126].

## Proteins involved in shaping the structure of internal membranes

### Cytoskeletal proteins

The shape of eukaryotic cells can be rather diverse, ranging from the simple structure of red blood cells to highly complex forms of neuronal cell. Bacteria can have simple structures, such as spheres or rods, but can also have more complex architectures such as spirals [129,130]. The cyanobacterial shapes vary from spheres (*Synechocystis*) or rods (*Synechococcus*) to filaments (*Anabaena sp. PCC 7120*, hereafter: *Anabaena*) [131].

The intracellular cytoskeleton is involved in shaping and stabilizing cell membranes in pro- and eukaryotes. While initially thought to have emerged in eukaryotes, the presence of prokaryotic homologs of eukaryotic cytoskeleton elements strongly suggests an ancient membrane organization system that has been further evolved in eukaryotes to the complex intramolecular system found these days [132]. While the cytoskeletal elements of eukaryotes can also serve as transport systems for motor proteins, only the static membrane-structuring function appears to have evolved early on in evolution.

Two classical eukaryotic cytoskeletal proteins are actin and tubulin, which form extended polymeric structures within cells. The globular actin proteins dynamically oligomerize into helical filaments in an ATP-dependent manner, whereas formation of rod-like microtubules from monomeric tubulins depends on GTP. The eukaryotic cytoskeletal elements are not only involved in shaping cells, cell motility, cell division, and intracellular trafficking, they also interact with intramembrane components and thereby organize the eukaryotic CM in defined diffusion areas, resulting, e.g., in hop diffusion events [133].

Both actin and tubulin have bacterial homologs. The dynamic (de)polymerization of the bacterial actin and tubulin homologs MreB and FtsZ is crucial for cell division and spore formation [134]. MreB (homologous) proteins are encoded in both bacteria and archaea [135–137]. As the eukaryotic actins, also the structurally and functionally similar bacterial actin homologous MreB and Mbl form filaments, which are, however, mostly nonhelical, and these proteins are involved in shaping bacterial membranes [132].

**Figure 4:**
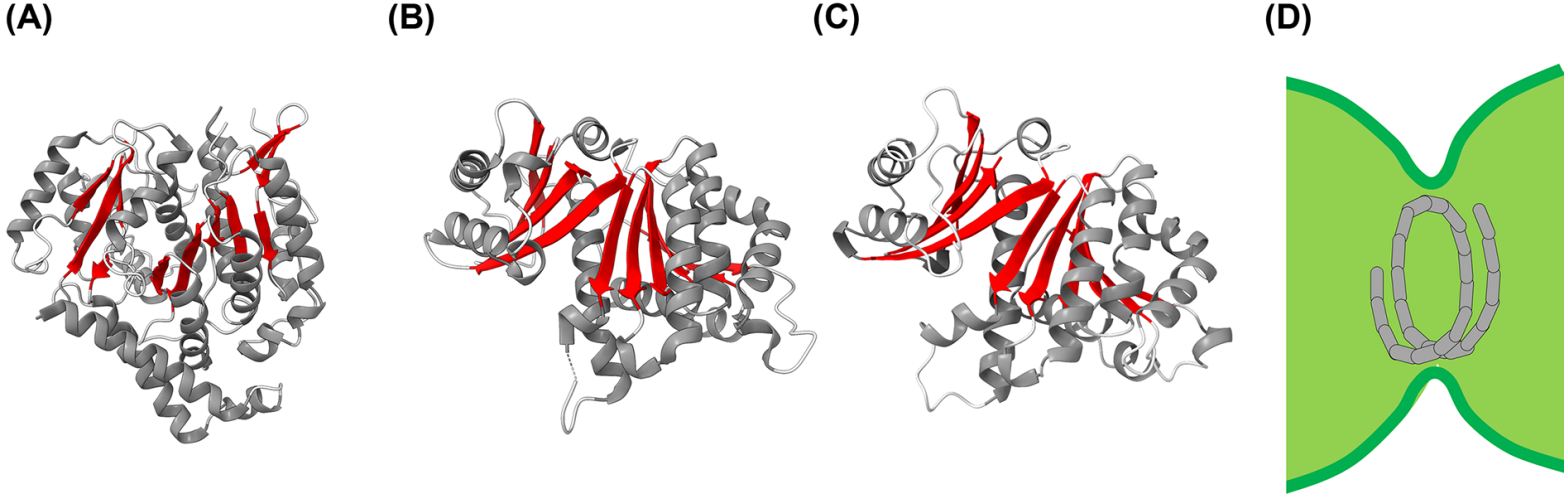
Structure and function of tubulin (homologs) (**A**) Structure of the human αTubulin (pdb 6E7B), the (**B**) *E. coli* FtsZ (pdb 6UNX), and (**C**) the AlphaFold model of the *Synechocystis* FtsZ protein (106,107). All proteins are rich in β-sheets. While the human Tubulin differs to some extent from the bacterial FtsZ proteins, the cyanobacterial and *E. coli* FtsZ appear to have a highly similar structure, likely due to their conserved function during cell division, where monomers form the membrane constricting FtsZ ring (**D**).

The bacterial homologs of tubulin are FtsZ, BtubA/B, and TubZ. Whereas FtsZ is present in almost all bacteria, BtubA/B and TubZ are only present in some species [138]. Although these proteins share only low sequence identity with tubulin, all proteins form polymers in a GTP-dependent process. The monomer structures of human tubulin, *E. coli* FtsZ and *Synechocystis* FtsZ are shown in Figure 4. FtsZ forms the Z-ring at the membrane that is essential for the constriction of the bacterial CM during cell division [139]. TubZ filaments are associated with the separation of replicated plasmids [140], whereas the function of BtubA/B proteins is unknown so far.

Also in cyanobacteria, actin and tubulin homologs are encoded, such as MreB and FtsZ [141]. MreB is present in almost all cyanobacteria and is essential in some cyanobacteria, such as *Synechococcus elongatus*, where it determines the cell shape [142]. Depletion of the *mreB* gene in the rod-shaped cyanobacterium *Synechococcus elongatus* results in spherical cells. Involvement in cell shaping is also reported for the filamentous cyanobacteria *Ananbaena* or *Fremyella diplosiphon* (also called *Calothrix sp. PCC 7601*) [141,143,144], yet MreB is not essential in spherical cyanobacteria, and even not encoded in *Synechocystis* [141].

The Z-ring-forming FtsZ protein is vital in all cyanobacteria investigated thus far, also in *Synechocystis* (Table 1) [141] and *Anabaena* [145]. Additional to the function in the divisome, also a role in cell–cell communication within filamentous cyanobacteria and the maintenance of their multicellularity has been suggested, since FtsZ seems to be necessary for the correct localization of the septal protein SepJ [146].

In summary, cyanobacterial proteins with homology to eukaryotic cytoskeletal elements are involved in membrane shaping with special importance in multicellular species.

### Proteins inducing and/or stabilizing curved membrane regions

High membrane curvature is important for the formation and dynamics of intracellular membrane systems in eukaryotes, and proteins stabilizing such highly curved membrane regions appear to be required. Examples of such interconnected membrane systems with curved membrane regions are the ER in eukaryotes or the TMs in chloroplasts or cyanobacteria.

The ER is composed of flat sheet-like, as well as highly branched tubular membrane networks. Its exact shape and size are highly dynamic and constantly regulated spatiotemporally in response to cellular (stress) conditions [147]. The generation of membrane curvature is an important step in the formation and dynamics of the ER membrane network. In the last decade, the family of reticulons has been identified as being involved in generating membrane curvature, typically seen as membrane tubulation *in vitro*, when these proteins are reconstituted with lipids [148–150]. The reticulons of different eukaryotic species, e.g., yeast *vs*. human, have only limited sequence identity, yet they all have a characteristic structure: all possess a conserved reticulon homology domain region of ∼200 amino acids, which consists of two transmembrane helical hairpin segments separated by a hydrophilic loop. The transmembrane helical hairpins appear to act as a wedge structure, which likely is responsible for the stabilization of highly curved membrane regions in the ER membrane [148,151].

A membrane organization of similar complexity as the ER is the TM found inside chloroplasts and cyanobacteria. Importantly, the highly curved chloroplast grana margins contain the protein CURT1 (Curvature thylakoid 1), which appears to control the size and exact number of grana discs [37,152]. In the plant *Arabidopsis thaliana*, four CURT1 proteins (CURT1A–D) are encoded, and the TM system in *A. thaliana curtABCD* deletions strains are essentially devoid of grana structures [152]. Generally, the grana structure of the TM system appears to correlate with the level of expressed CURT1 proteins [152].

**Figure 5:**
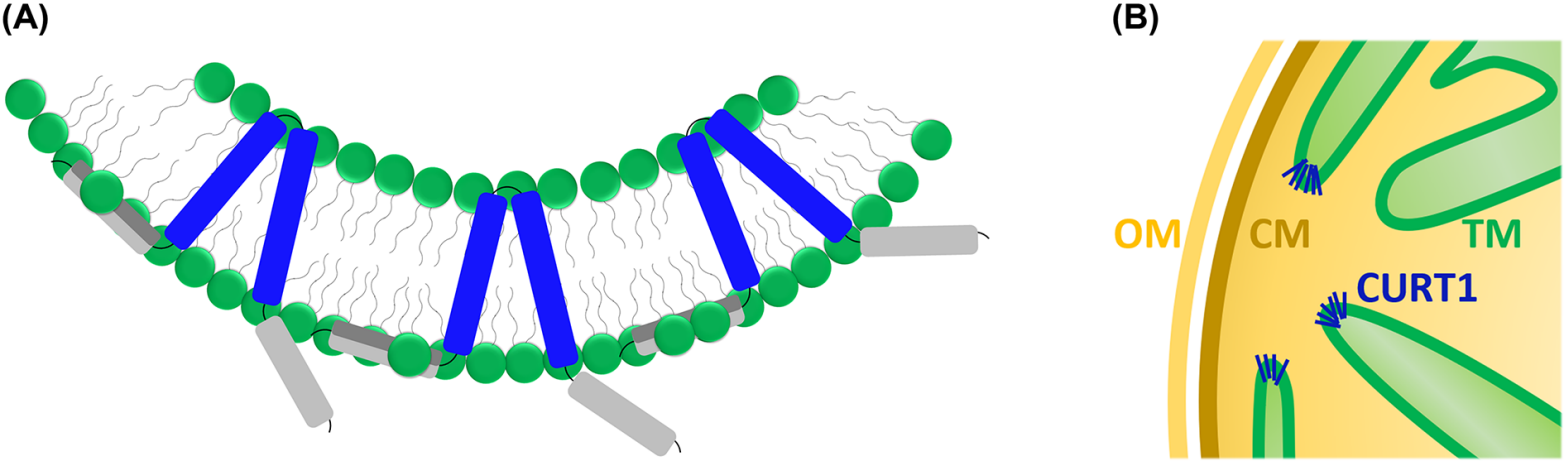
Putative structure and function of the *Synechocystis* CURT1 protein (**A**) The CURT1 protein is predicted to have four α-helices (106,107), two of which are membrane-spanning and two are soluble. The transmembrane helices are wedge shaped, which likely results in membrane curvature formation. The first helix is predicted to be amphipathic, and membrane interaction of this helix might (additionally?) curve membranes. (**B**) CURT1 proteins appear to accumulate at highly curved TM regions within the cyanobacterial TM network and thereby induce and/or stabilize high-curved TM margin regions.

Also in cyanobacteria, CURT1 homologs are encoded, albeit cyanobacteria do not contain grana TMs [153]. Still, the TM systems of, e.g.,* Synechocystis* contain highly curved convergence zones, which may act as CM-TM contact sites [38]. Interestingly, species such as *Prochlorococcus marinus PCC 9511* (hereafter: *Prochlorococcus*) or *Synechococcus elongatus*, which do not contain convergence zones, do not encode CURT1 homologs [153]. Deletion of the CURT1-coding gene in the cyanobacterium *Synechocystis* resulted in a disturbed organization of the TM system completely lacking any curved membrane region [153]. While the structures of *Arabidopsis* CURT1 or *Synechocystis* CURT1 have not been solved yet, these are predicted to consist of two transmembrane helices and two cytoplasmic helices [152], of which the N-terminal helix is putatively amphipathic (Figure 5). This structure corresponds to about half of a classical reticulon, and CURT1 proteins thus appear to be small representatives of membrane-bending/shaping proteins (a ‘semireticulon’). Oligomerization of CURT1 proteins has been suggested to play a role for the curvature-inducing function, and CURT1 proteins are discussed to be necessary during TM biogenesis [154,155]. Likely, the wedge-shape structure of the transmembrane helical hairpins induces curvature, as suggested for reticulons [156]. Yet, the first formed α-helix in CURT1 is amphipathic, and membrane adhesion of amphipathic helices is well known to sense and/or induce membrane curvature [157,158]. Thus, it currently is elusive whether the N-terminal amphipathic helix or the transmembrane helical hairpin alone are crucial for induction of membrane curvature, or whether membrane curvature is induced by the synergistic action of the transmembrane helical hairpin plus the amphipathic helix.

## Proteins involved in dynamic membrane remodeling

Besides organizing and/or shaping the structures of internal membranes, proteins can also actively disturb the membrane structure, eventually resulting in membrane destabilization. To do so, proteins need to perturbate the lipid bilayer structure either by (at least partial) insertion of the protein into the membrane (hairpins, loops, transmembrane helices), or by scaffolding effects upon membrane binding. The interactions finally result in a membrane-destabilizing structure, such as formation of a protein-stabilized pore, or in membrane fusion or fission. In fact, in eukaryotes, gasdermines form large membrane pores in response to pathogens or toxins, a mechanism of defense that typically results in cell death. Furthermore, eukaryotic cells require membrane-remodeling proteins to conduct physiological processes, such as endocytosis, exocytosis, membrane fusion and fission, and membrane repair. The mechanisms mediating membrane remodeling and the formation of vesicles have been intensively studied in eukaryotic systems, for instance, in the context of the secretory pathway. The existence of intracellular vesicles within prokaryotic cells still is under debate, albeit in some bacteria and archaea air-filled gas vesicle are observed as a hollow structure made of protein [159]. These intracellular vesicles are involved in vertical migration of aquatic microbes [160]. Yet, these structures are not further discussed here, since these vesicles are not membrane-coated. In contrast, outer membrane vesicles (OMVs) have been observed in many Gram-negative bacteria including the cyanobacterium *Synechocystis* [161]. Since we focus on the dynamics of inner membranes, we here only refer to other articles describing OMV biogenesis [161,162].

Membrane fission/fusion proteins might be involved in remodeling and/or repair of the CM, as well as in TM dynamics in cyanobacteria [163]. In fact, membrane remodeling, involving membrane fusion and fission events, has been observed in chloroplast TMs [164], and likely dynamin-like proteins (DLPs) are involved. Besides membrane remodeling, it clearly is also crucial for survival of bacterial cells that cellular membranes are intact, for example, to ensure that exclusively gated transport of molecules and ions across membranes occurs. Thus, efficient membrane repair mechanisms, that involve membrane fusion and/or fission [165], are indispensable also in bacteria if a cell has to cope with membrane damage. Recently, proteins of the ESCRT-III superfamily have been identified in bacteria, and these proteins can stabilize membranes and/or mediate membrane dynamics, involving membrane repair. While some homologs of proteins that are involved in the secretory pathway in eukaryotes have been identified in chloroplasts [105,166–169] and also cyanobacteria [167], currently the exact physiological functions of these proteins are enigmatic and it still is unclear whether vesicle transfer processes exist in chloroplasts and/or cyanobacteria. Thus, we here refrain from further discussing these factors and refer to other articles [170,171].

### Transmembrane pore-forming proteins

While pro- and eukaryotic cells have evolved mechanisms to stabilize and repair damaged membranes, in some cases a rather drastic form of membrane remodeling is observed, i.e., the formation of large membrane pore structures. While regulated membrane pore formation might allow selective transmembrane diffusion of molecules when the formed pore has a selectivity filter, formation of large, unselective pores results in disruption of the membrane potential, and finally in cell death. Prominent examples of such pore-forming proteins are antimicrobial peptides, such as Nisin of *Lactococcus lactis* [172,173], or pore-forming toxins, such as α-hemolysin of *Staphylococcus aureus* [174,175].

**Figure 6:**
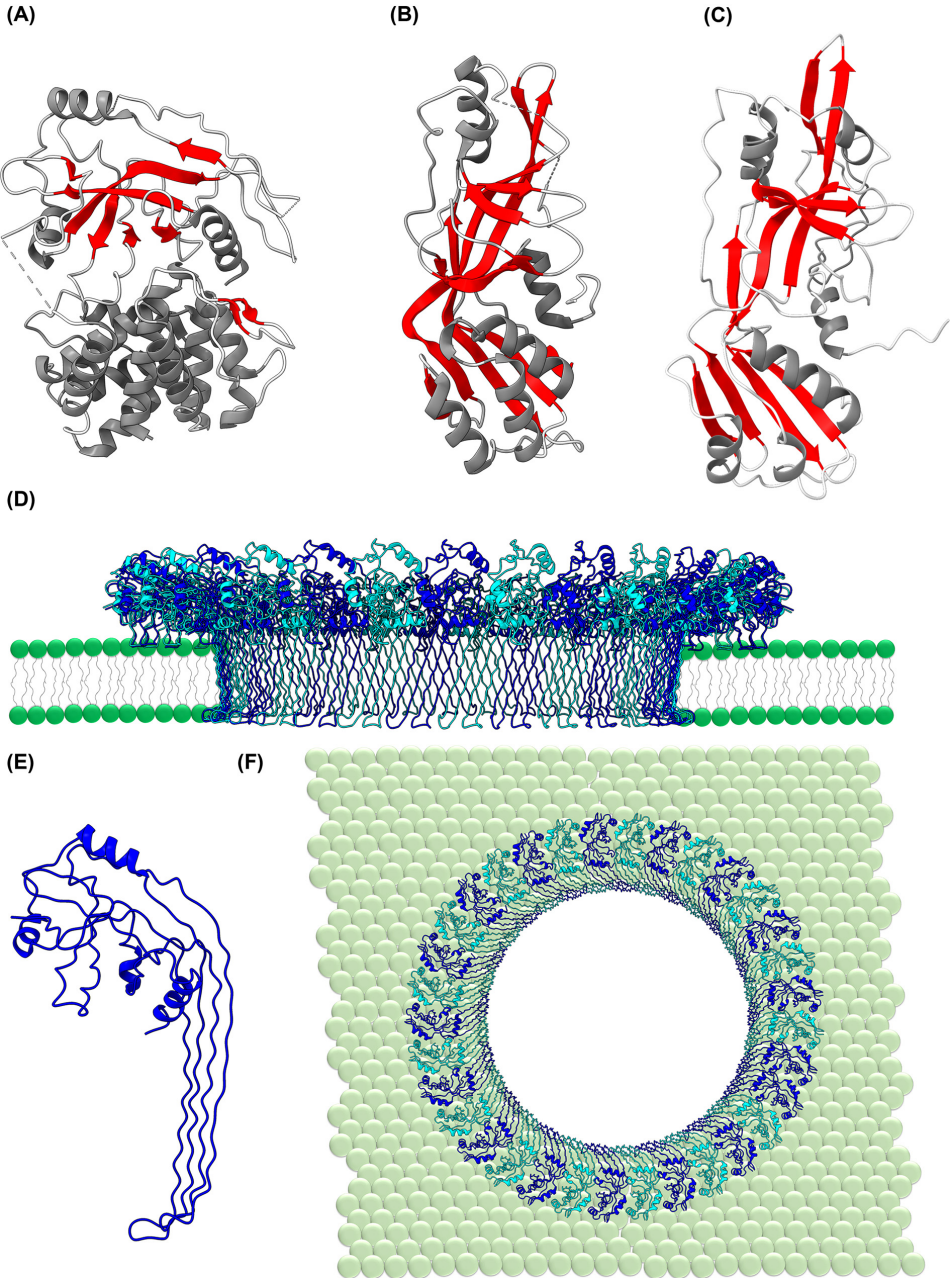
Structure and function of eu- and prokaryotic gasdermins Structure of (**A**) the monomeric human GSDM-D (pdb 6N90), (**B**) a bacterial GSDM (of *Bradyrhizobium tropiciagri*, pdb 7N50), and (**C**) the AlphaFold predicted structure of a cyanobacterial GSDM (of *Nostoc* sp. NIES-2111) [106,107]. The proteins have an extended β-sheet structure, which reorganizes after cleavage of an inhibitory peptide to form an elongated monomer (**E**) that forms large, homo-oligomeric rings (**D,F**). The oligomeric ring partially integrates into and forms a pore within the membrane. The structure of the human GSDM-D ring (pdb 6VFE) is shown in a side view (**D**) as well as when embedded within a membrane model (**F**).

In eukaryotes, gasdermines (GSDMs) oligomerize into large β-barrel membrane pores in response to pathogens or toxins [176–178] (Figure 6). The GSDM family is conserved in mammals, fungi, and also in bacteria, as reported only very recently [179,180]. The auto-inhibited form of GSDMs is usually located in the cytosol, and cleavage of an N-terminal domain, mediated by caspases or caspase-like proteins, releases the active C-terminal GSDM domain. In eukaryotes, proteolytic activation is triggered by bacterial infections or bacterial toxins. In mammalian cells, GSDM membrane pore formation ultimately leads to pyroptosis, an inflammatory form of lytic-programmed cell death [180]. GSDM-D is the thus far best-studied protein of the human GSDM family, which contains six GSDMs in total. GSDM-D specifically binds to negatively charged lipids, such as phosphatidylinositol phosphates at the inner leaflet of the CM or to cardiolipin of the bacterial membrane, and it has been suggested that soluble GSDMs monomers might pass GSDM pores formed in an eukaryotic CM, bind to bacterial membranes and there again form pores as further pathogen defense. Since phosphatidylinositides are distributed asymmetrically in the CM bilayer, GSDMs likely do not bind to the outer membrane leaflet of adjacent cells, which prevents extensive tissue damage. In general, membrane binding induces the oligomerization of the activated GSDM-D to form a pore of about 12–15 nm, which causes dissipation of the membrane potential and finally cell swelling and lysis. Besides the lytic functions, also nonlytic functions have been reported [181]. Here, cytokines get released through the pore and induce a rapid innate immune response. Since dynamic opening and closing of the pores are suggested, the release of cytokines may even be intrinsically regulated [182]. The repair of the pore-containing membrane regions is suggested to be mediated by vesicle abscission into the extracellular space mediated by ESCRT complexes, which is discussed below.

Recently, genes coding for bacterial GSDMs (bGSDMs) have been detected in bacterial genomes [180,183] (Figure 6). In bacteria, a bacteriophage infection activates the GSDMs via cleavage of an inhibitory peptide, as observed in eukaryotic systems, albeit the inhibitory peptide is distinctly smaller than the eukaryotic inhibitory domain. The size of the bGSDM pores seems adaptable for the secretion of different molecules [177,184]. Yet, the observed pores were mostly larger (20–30 nm) than the pores formed by the eukaryotic GSDM-D (12–15 nm). The recently discovered evolutionary conservation strongly suggests that GSDM-mediated pore formation is actually an ancient component of the innate immune system in both eu- and prokaryotes.

Potential bGSDM-coding genes have also been identified in some cyanobacterial genomes, such as *Nostoc* sp. NIES-2111, and thus GSDM pore formation is a conserved mechanism that appears to be of importance in at least some cyanobacteria [180]. In these organisms, the GSDM-mediated membrane destabilization likely also is a reaction on a phage infection. Yet, as cyanobacteria contain two separated internal membrane systems, the TM and the CM, the question arises as to how cyanobacterial bGSDMs specifically recognize the CM. As TMs offer far more surface for interaction with bGSDM monomers, the probability of pore formation within the TM system is substantially higher, which clearly results in destabilization and disruption of the TM structure and function. Yet, as bGSDM pore formation triggers cell death, TM destabilization might just be tolerated as a side effect and formation of a few pores within the cyanobacterial CM is sufficient. Nevertheless, membrane specificity and the exact physiological function of bGSDMs in cyanobacteria still need to be elucidated.

### Membrane fusion and/or fission mediated by DLPs

The dynamin superfamily is involved in fission and fusion of membranes in both eu- and prokaryotes. In eukaryotes, dynamins are involved in various cellular functions, such as endocytic vesicle fission, intracellular trafficking, mitochondrial fission and fusion, peroxisomal fission, ER tubule fusion, chloroplast division, and cytokinesis [185]. All proteins of the dynamin superfamily share a modular structure (G-domain, BSE (bundle signaling element), and a stalk domain), a large size (>70 kDa) and self-assemble to large oligomeric structures, which act as mechanoenzymes [186]. In contrast with classical GTPases of the Ras or Ran family, the nucleotide-binding affinity is rather low, albeit the basal GTPase activity is high. Typically, the basal activity of dynamins is further stimulated upon oligomerization or upon binding to appropriate membranes.

DLPs are classified as fission DLPs, fusion DLPs, and membrane-independent scaffold DLPs [185]. While fission DLPs are soluble proteins that reversibly bind to membranes, fusion DLPs are typically membrane-anchored. Representative examples of fission DLPs are mammal Dyn1–3, mammal DRP1, and yeast Dnm1, which are involved in the fission of clathrin-coated vesicles, mitochondria, or peroxisomes, respectively [187,188]. Typical examples of fusion DLPs are the mammal mitofusins 1/2 (Mfn1/2) or alastins (ATL1–3), which act on the mitochondrial outer membrane or the ER membrane, respectively [189,190]. Yet, some DLPs are reported to have both, fusion and fission activities, such as the optic atrophy 1 (OPA1) or the mitochondrial protein genome maintenance 1 (Mgm1p) [191,192]. Examples of eukaryotic membrane-scaffolding DLPs are Mx proteins (Myxovirus resistance A/B) or guanylate-binding proteins (GBPs) [193,194].

**Figure 7:**
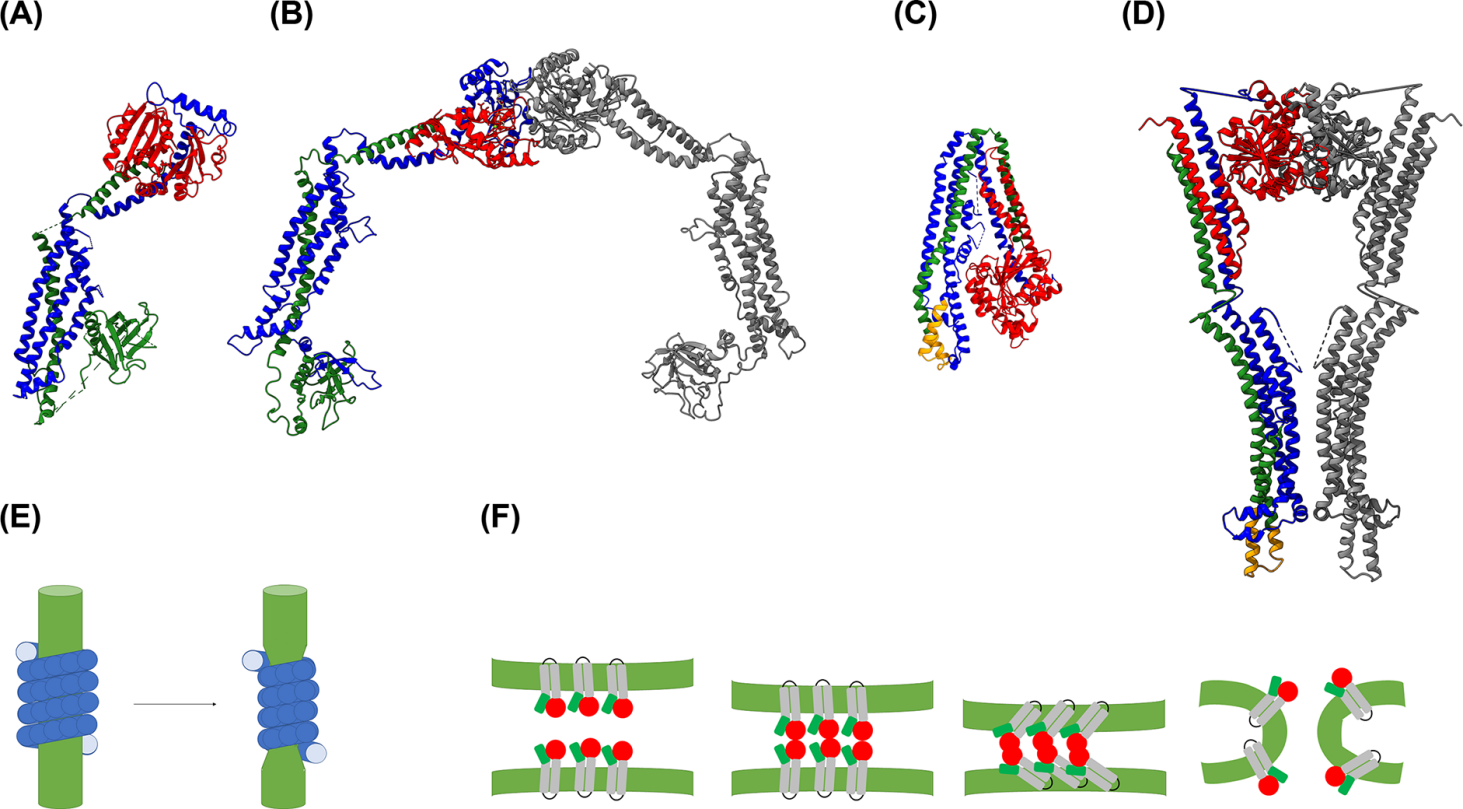
Structure and function of DLPs The structures of the human Dynamin in (**A**) absence (pdb 3SNH) and (**B**) presence of membranes (pdb 6DLU), as well as the structures of the cyanobacterial bDLP1 of *Nostoc punctiformae* (**C**) in its inactive state, i.e., in absence of a membrane (pdb 2J69), and (**D**) the membrane-bound active state (pdb 2W6D) are shown. Dynamin and DLPs trigger membrane fission or fusion events. (**E**) During membrane fission, Dynamin forms a spiral around a membrane neck, which constricts upon GTP hydrolysis. (**F**) The mechanism of membrane fusion mediated by DLPs is not completely understood. Possibly, oligomerization of DLPs attached to adjacent membranes finally tethers two membranes. Structural rearrangements upon GTP hydrolysis eventually enable the fusion of adjacent membranes.

The mechanism by which eukaryotic dynamins trigger membrane fission is largely resolved (Figure 7). The proteins dimerize via the G-domain and further oligomerize into large, helical structures on a membrane surface. The final fission of the membrane is then induced by the constriction of the helical assembly around a tubular membrane neck upon GTP hydrolysis. While dynamin mediates complete membrane fission [195], the membrane deformation caused by Drp1 or OPA1 may be restricted to membrane constriction [185]. In case of membrane fusion DLPs, the proteins may form a planar lattice between two bilayers, which tethers membranes closely to enable fusion. A small hydrophobic region is discussed to insert into the membrane and thereby spatiotemporarily perturbates the membrane structure, further increasing the membrane fusion propensity. A different mechanism has also been suggested, where GD-dimerization of adjacent DLPs in the membrane triggers nucleotide-dependent membrane fusion (e.g., Alastin, Mitofusion), and Mgm1 might even first tubulate membranes followed by fusion of the tubules [196,197].

About two decades ago, bacterial DLP (bDLP)-coding genes have been identified also in bacterial genomes [198], and a bDLP has been identified in the cyanobacterium *Nostoc punctiforme PCC 73102* (hereafter: *Nostoc*) [199] (Figure 7). As their eukaryotic counterparts, bDLPs are also reported to be involved in membrane-remodeling processes, involving membrane fusion/fission events. Yet, while the eukaryotic dynamin is studied intensively, only limited information about bDLPs is available. Examples of bDLPs are the membrane fusion protein *B. subtilis* DynA [200,201], the chromosome partitioning CrfC of *E. coli*, the OMV-related LeoA of *E. coli*, the membrane fission protein IniA of *Mycobacterium tuberculosis* [202] or the membrane fusion DLPs 1/2 of *Campylobacter jejuni* [203]. Noteworthy, the membrane fusion activity of the thus far best-studied bDLP DynA does not depend on GTP hydrolysis *in vitro*, and LeoA did not show GTPase activity at all [204,205]. The proteins DynA and DynB of *Streptomyces venezuelae* are reported to interact with FtsZ and thereby mediate cell division or the formation of sporulation septa [206]. bDLPs are thus far the only recognized membrane-remodeling enzymes where membrane remodeling is coupled to GTP hydrolysis in bacteria, emphasizing their potential involvement in large membrane-remodeling processes [4]. For a long time, the *in vivo* function of bDLPs was not clear, and only recently a relevance during phage infections has been identified as a novel resistance mechanism [207]. While phage infection is not inhibited by DynA, the cell lysis following phage infection was delayed, preventing fast spreading of the phages. DynA forms large clusters at membranes, and thus, likely stabilizes the bacterial membranes, which might even involve repair of damaged membrane regions via the *in vitro* observed GTP-independent membrane fusion activity [201,207].

bDLPs are encoded in many bacterial species, sometimes even multiple family members, as has been observed in several cyanobacterial strains [208]. Some bacteria, such as *C. jejuni*, even encode multiple potential bDLPs, which might form hetero-oligomers [203]. An example of a cyanobacterial bDLP is the ‘bDLP1’ of the cyanobacterium *Nostoc*. While its molecular function is not clear thus far, *Nostoc* DLP binds to membranes, resulting in formation of membrane tubules *in vitro*, a behavior typically observed with eukaryotic fission DLPs [209]. Recently, a bDLP has also been identified in the genome of the cyanobacterium *Synechocystis* (Slr0869) (Table 1) [208]. The isolated protein has typical dynamin-like features and mediates membrane fusion independent of nucleotides, albeit the proteins oligomerize into a structure typical for eukaryotic fission dynamins [210]. The DLP is expressed in *Synechocystis* where it interacts with the negatively charged TM lipids SQDG or PG. It has been suggested that the *Synechocystis* DLP is involved in dynamics/repair of the TMs at stress conditions [210].

In summary, DLP-mediated membrane remodeling likely is relevant in cyanobacteria at all conditions that require large energy-dependent membrane-remodeling processes, whereby the energy of nucleotide hydrolysis is converted into mechanic membrane deformation. These processes likely include light-dependent TM rearrangements but also remodeling in response to nutrient limitations or stress induced, e.g., by phage infections.

### Membrane stabilization, fission and/or fusion mediated by ESCRT-III proteins

Several eukaryotic membrane-remodeling processes, such as cytokinesis [211], multivesicular body (MVB) formation [212], or endosomal fission, are mediated by the highly conserved *endosomal sorting complexes required for transport* (ESCRT) [213]. The ESCRT machinery consists in eukaryotes of the five protein complexes, ESCRT-0, ESCRT-I, ESCRT-II, ESCRT-III, and the AAA-ATPase Vps4. Of these complexes, ESCRT-III is the core component mainly responsible for the membrane-remodeling activity.

**Figure 8:**
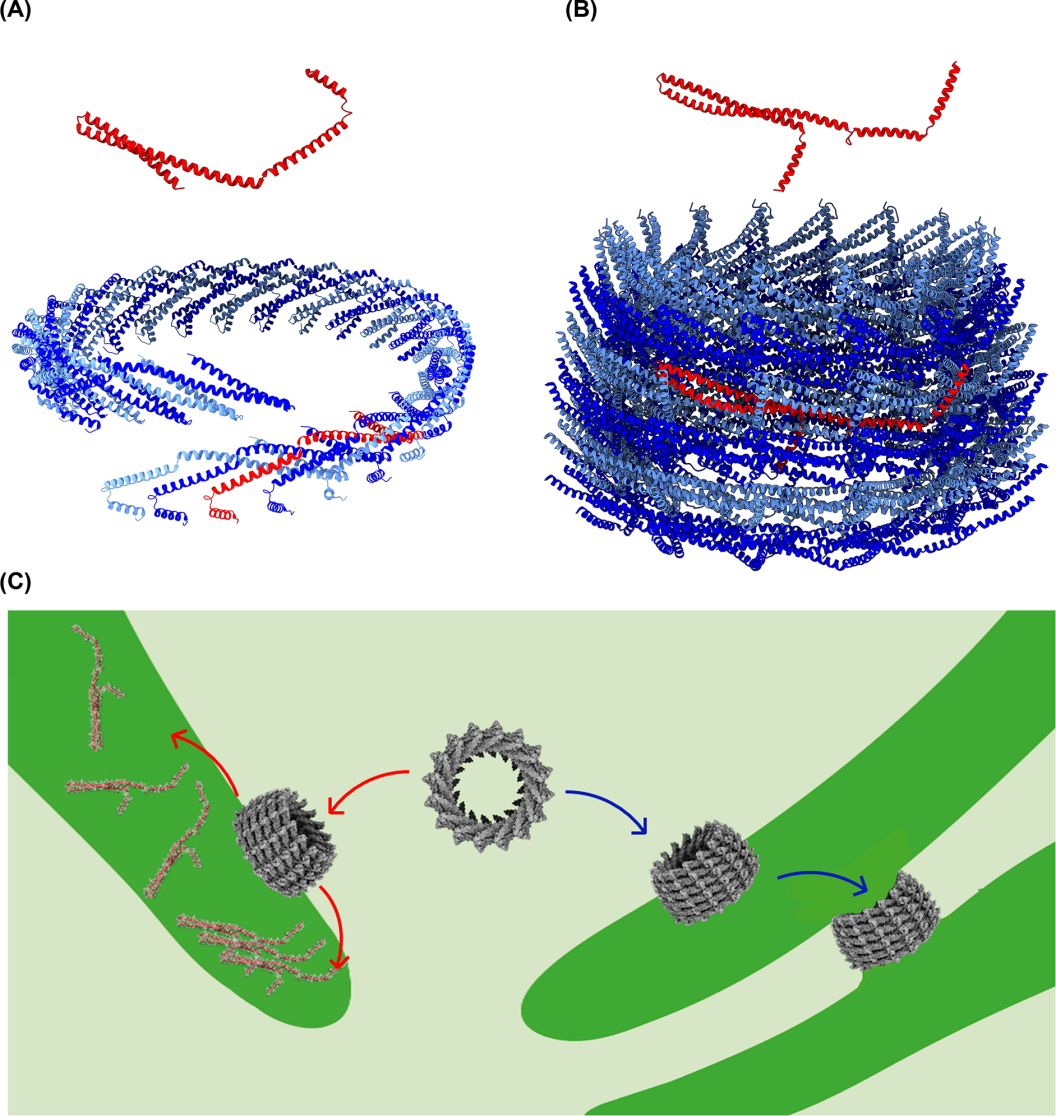
Membrane remodeling mediated by proteins of the ESCRT-III superfamily The ESCRT-III proteins (**A**) human CHMP1B (pdb 6TZ9) and (**B**) IM30 of *Synechocystis* (pdb 7O3Y) have similar secondary structures, with a central coiled-coil. The monomers oligomerize into large spirals (CHMP1B, pdb 6TZ9) or rings (IM30, pdb 7O3Y), which are essential for membrane remodeling. (**C**) In cyanobacteria, IM30 likely has a dual function: Membrane protection involves disassembly of the ring on the membrane surface and formation of a membrane-covering carpet structure. This structure might protect cyanobacteria against proton loss at damaged TM regions. The membrane fusion activity involves binding to the membrane as a ring, which further engulfs membranes.

Interaction of the ESCRT complex with a membrane leads to membrane deformation and scission from the luminal side of the membrane. Membrane deformation is mediated by oligomerization of ESCRT-III subunits into large, spiral-forming supercomplexes on the membrane (Figure 8). In eukaryotes, more than four core ESCRT-III subunits exist [214], which assemble into hetero-oligomeric (active) polymers [215], albeit the ESCRT-III protein Snf7 can induce membrane deformation already without further ESCRT-III proteins, at least *in vitro* [216]. Nevertheless, while all eukaryotic ESCRT-III proteins share a common core structure of five α-helices (Figure 8), each subunit appears to contribute a specific function to the filamentous polymer. Assembly of individual ESCRT-III proteins in the right order appears to be crucial for membrane remodeling *in vitro* [217].

In recent years, ESCRT-III homologous have been detected both in archaea [218] and bacteria (PspA/IM30 family) [219–221], and members of the ESCRT-III superfamily are therefore conserved in all kingdoms of life.

The bacterial PspA and the IM30 (also known as the Vipp1) adopt a canonical ESCRT-III-fold (Figure 8). While the sequence identity is not high, the structure of the IM30/PspA and ESCRT-III is very similar, with five conserved α-helical regions, from which two form a long coiled-coil hairpin structure. Similar to the eukaryotic superfamily members, also bacterial ESCRT-IIIs form large oligomeric super complexes and bind to as well as remodel membranes, yet, bacterial ESCRT-IIIs form homo- rather than hetero-oligomeric structures.

The function of the IM30/PspA family members clearly is related to membrane dynamics. PspA was initially identified in *E. coli* cells following infection with filamentous phages [222]. It has subsequently been shown that PspA is a member of a bacterial stress response system, referred to as the ‘phage shock protein system’ (Psp-system). A common denominator of all stress conditions appears to be the reduction of the proton motif force across the bacterial CM [223–225].

The thus far best-studied Psp-system of *E. coli* comprises a total of seven *psp* genes [226]. While the Psp-system appears to be wide-spread throughout all bacterial domains, phylogenetic analyses indicate that PspA homologs are present in organisms without connection to any other component of the Psp-response, suggesting either a strong reduction of the Psp-system or a newly acquired function of the remaining PspA-homolog during evolution [227–229]. In fact, PspA is the only member of the bacterial Psp system that is conserved in bacteria [228]. It is assumed that PspA supports bacterial cells in stress situations via membrane binding and blocking proton leakage or via inducing the down-regulation of the proton motif force-consuming processes [226,230]. It is assumed that PspA forms large, scaffolding oligomers upon binding to negatively charged membrane surfaces [231]. LiaH, a PspA-homolog of the Gram-positive bacterium *B. subtilis*, belongs to the Lia-system that is conserved in all *Firmicutes*, *Bacillus*, and *Listeria* species, where it regulates the bacterial stress response [232–234]. While most components of the Psp- and Lia-system differ, the primary target proteins of both systems, LiaH and PspA, show sequence and structural homology, suggesting a similar mode of action [233,235].

IM30 is a bacterial member of the ESCRTIII-protein family, which is conserved in oxygenic phototrophic organisms (cyanobacteria and chloroplasts). Although sequence identity is not high between PspA and IM30 proteins [236], the recently solved structures of two IM30 and a PspA protein demonstrate that the protein structures are highly similar with an N-terminal core structure of about 220 amino acids [219–221]. A major structural difference between PspA and IM30 is an additional C-terminal α-helix in IM30 proteins that is connected to the PspA domain via an extended linker region [237,238].

IM30 likely has evolved from PspA via gene duplication [237]. In cyanobacteria, both PspA and IM30 are encoded [227], even though PspA cannot substitute the vital IM30 function *in vivo* [239]. As PspA, also IM30 is linked to membrane maintenance/repair under stress conditions [240,241], and furthermore to membrane remodeling by triggering membrane fusion events (reviewed in [242,243]) (Figure 8). Membrane fusion and membrane protection are in part contradicting, as membrane fusion requires at least partial destabilization of the bilayer structure. Consequently, spatiotemporal separation of the IM30 functions is necessary. Currently, Mg^2+^-binding and/or phosphorylation have been suggested to differentially regulate the IM30 activity [55,244].

While a membrane-remodeling activity has initially been assumed to be a unique function of IM30s, PspA can also trigger membrane fusion and/or fission, at least *in vitro* [221]. Nevertheless, the membrane-protective activity of PspA is established, whereas the physiological relevance of PspA-mediated membrane remodeling still remains to be shown.

The molecular mechanisms mediating and regulating PspA/IM30-membrane interactions are not understood in detail yet. The membrane-protective and the membrane-remodeling functions probably have different modes of action, albeit a mechanism of membrane repair, involving fusogenic events, has been suggested recently [241]. *In vitro*, the formation of large IM30 carpet structures has been observed on model membranes, which appear to block proton leakage, similar to PspA [230,240]. These carpets could resemble IM30 assemblies at the TM, which were observed *in vivo*, at light-stressed membranes [245,246]. The large oligomeric IM30 rings need to disassemble upon membrane adhesion to form the flat carpet structures [240]. In presence of Mg^2+^, IM30 instead binds to membrane surfaces as a ring, and upon surface adhesion of IM30 rings, membrane pores form, at least *in vitro* [244], which might be the basis for the observed membrane destabilizing and fusion activity [247]. Detailed *in vitro* analyses now suggest that PspA and IM30 rings/rods partly engulf membranes and thereby allow membrane remodeling [220,221,248], and an IM30 ring engulfing a membrane has probably also been observed *in vivo* [220].

While we just begin to understand the cellular functions of IM30, the protein clearly is one key component crucial for TM biogenesis. Yet, besides the discussed membrane fusion and protection activities, many more functions have been suggested in the past, involving the transport of lipids or proteins from the CM to the TM [249–251], the insertion of TM proteins [252,253] or vesicle formation [254]. Most functions, such as vesicle formation or lipid/protein transport, can be explained by the now observed membrane-remodeling activity, at least in part. IM30-induced TM fusion likely is crucial during TM biogenesis and (re)development of the TMs, e.g., after dark-to-light transition or nutrient limitation, which can lead to almost complete degradation of the TM system [55,245,246,251,255]. Here, membrane-remodeling processes are absolutely vital and clearly include membrane fusion processes [57].

## Concluding remarks

The here discussed examples of evolutionarily conserved proteins involved in (cyano)bacterial membrane dynamics strongly suggest that the proteins and protein families are no eukaryotic inventions but initially appeared in prokaryotes, albeit the proteins typically have gained more complex functions in eukaryotes. As biochemical *in vitro* analyses of a protein’s structure and function, using purified proteins, often is challenging when analyzing the eukaryotic proteins, studying the structure, dynamics, and activity of the bacterial homologs can clearly help to unravel and/or better understand also their molecular mode of action, as the bacterial homologs often have less complex structures. Nevertheless, transferring these principles to the eukaryotic system has to be done with great care and may not be possible in all cases. However, currently the bacterial proteins are typically studied less intensively than the human homologs. Yet, the identification of bacterial homologs of eukaryotic membrane remodelers within various (cyano)bacterial species clearly was and is an important step to better understand bacterial membrane dynamics. Obviously, several general principles guiding membrane dynamics have already evolved in prokaryotes and were transferred to and further developed in eukaryotic cells. Yet, while cyanobacterial proteins (putatively) involved in membrane dynamics have now been identified and involvement of these proteins in TM dynamics has been analyzed to some extent, their interconnection and the regulation of their respective activities is essentially not understood. Taking, e.g., into account the evolutionary relation of the eukaryotic ESCRT-III and the bacterial IM30/PspA proteins, which has been detected only very recently based on the structures of the bacterial proteins, we are optimistic that more examples of membrane remodelers conserved in eukaryotes and bacteria will be discovered in the near future.

## Abbreviations

ABC: ATP-binding cassette
bDLP: bacterial DLP
bGSDM: bacterial GSDM
BSE: bundle signaling element
CM: cytoplasmic membrane
CURT1: curvature thylakoid 1
DGDG: digalactosyldiacylglycerol
DLP: Dynamin-like protein
ER: endoplsmic reticulum
ESCRT: endosomal sorting complexes required for transport
GBP: guanylate-binding protein
GD: GTP-binding domain
GSDM: gasdermine
IE: inner envelope
IM30: inner membrane-associated protein of 30 kDa
MGDG: monogalactosyldiacylglycerol
MVB: multivesicular body
OMV: outer membrane vesicle
OPA: optic atrophy 1
PE: phosphatidylethanolamine
PG: phosphatidylglycerol
PSI: photosystem I
PspA: phage shock protein A
SQDG: sulfoquinovosyldiacylglycerol
TM: thylakoid membrane
Vipp1: vesicle-inducing protein in plastids 1
